# Gene Silencing and Haploinsufficiency of *Csk* Increase Blood Pressure

**DOI:** 10.1371/journal.pone.0146841

**Published:** 2016-01-11

**Authors:** Hyeon-Ju Lee, Ji-One Kang, Sung-Moon Kim, Su-Min Ji, So-Yon Park, Marina E. Kim, Baigalmaa Jigden, Ji Eun Lim, Sue-Yun Hwang, Young-Ho Lee, Bermseok Oh

**Affiliations:** 1 Department of Biochemistry and Molecular Biology, School of Medicine, Kyung Hee University, Seoul, Korea; 2 Department of Physiology, College of Medicine, Yonsei University, Seoul, Korea; 3 Department of Chemical Engineering, College of Engineering, HanKyong National University, Ansung-Si, Gyeonggi-Do, Korea; University of Utah School of Medicine, UNITED STATES

## Abstract

**Objective:**

Recent genome-wide association studies have identified 33 human genetic loci that influence blood pressure. The 15q24 locus is one such locus that has been confirmed in Asians and Europeans. There are 21 genes in the locus within a 1-Mb boundary, but a functional link of these genes to blood pressure has not been reported. We aimed to identify a causative gene for blood pressure change in the 15q24 locus.

**Methods and Results:**

*CSK* and *ULK3* were selected as candidate genes based on eQTL analysis studies that showed the association between gene transcript levels and the lead SNP (rs1378942). Injection of siRNAs for mouse homologs *Csk*, *Ulk3*, and *Cyp1a2* (negative control) showed reduced target gene mRNA levels *in vivo*. However, *Csk* siRNA only increased blood pressure while *Ulk3* and *Cyp1a2* siRNA did not change it. Further, blood pressure in *Csk*^+/-^ heterozygotes was higher than in wild-type, consistent with what we observed in *Csk* siRNA-injected mice. We confirmed that haploinsufficiency of *Csk* increased the active form of Src in *Csk*^+/-^ mice aorta. We also showed that inhibition of Src by PP2, a Src inhibitor decreased high blood pressure in *Csk*^+/-^ mice and the active Src in *Csk*^+/-^ mice aorta and in *Csk* knock-down vascular smooth muscle cells, suggesting blood pressure regulation by Csk through Src.

**Conclusions:**

Our study demonstrates that *Csk* is a causative gene in the 15q24 locus and regulates blood pressure through Src, and these findings provide a novel therapeutic target for the treatment of hypertension.

## Introduction

Blood pressure is influenced by a variety of mechanisms that involve many genetic factors. To detect genetic markers for blood pressure, genome-wide association studies (GWASs) have been performed using large human samples from various ethnic groups and have identified many genetic loci that are associated with blood pressure and hypertension. The Korean Association REsource (KARE) [1], the Global Blood Pressure Genetics (GlobalBPgen) [2], Cohorts for Heart and Aging Research in Genome Epidemiology (CHARGE) [3], the Asian Genetics Epidemiology Network Blood Pressure (AGEN-BP) [4], and International Consortium for Blood Pressure (ICBP) [5] have conducted GWASs on blood pressure and hypertension, identifying 33 independent loci that have reached a genome-wide significance level.

The 15q24 locus is significantly associated with blood pressure in Asians and Europeans, as reported by Global BPgen [2] (rs1378942, *P* = 2×10^−6^ with systolic blood pressure (SBP), *P* = 6×10^−8^ with diastolic blood pressure (DBP), N = 34,433 Europeans), the CHARGE consortium [3] (rs6495122, *P* = 2.7×10^−5^ with SBP, *P* = 1.8×10^−7^ with DBP, N = 29,136 Europeans), AGEN [4] (rs1378942, *P* = 6.5×10^−6^ with SBP, *P* = 1.0×10^−5^ with DBP, N = 41,447 Asians), and ICBP [5] (rs1378942, *P* = 5.7×10^−23^ with SBP, *P* = 2.7×10^−26^ with DBP, N = 69,395 Europeans). This link has also been confirmed by Takeuchi et al. [6] (rs1378942, *P* = 0.05 with SBP, *P* = 0.009 with DBP, N≤24,300 Japanese), Tabara et al. [7] (rs1378942, *P* = 0.007 with SBP, *P* = 0.015 with DBP, N = 13,920 Japanese), Hong et al. [8] (rs1378942, *P* = 2.48×10^−5^ with SBP, *P* = 4.58×10^−5^ with DBP, N = 8,842 Koreans), and Ganesh et al. [9] (rs7085, *P* = 6.68×10^−11^ with SBP, *P* = 7.936×10^−11^ with DBP, N = 61,619 Europeans).

In the 15q24 locus, there are at least 21 genes near the lead SNP (rs1378942) within a 1-Mb boundary. Among these genes, several Expression Quantitative Trait Loci (eQTL) analysis studies have shown that the expression levels of *CSK* (c-src tyrosine kinase) and *ULK3* (unc-51-like kinase3) are significantly associated with polymorphism of rs1378942 in blood, lymphoblastoid cell lines (LCLs), and monocytes (*CSK*, *P* = 1.97×10^−45^, *P* = 1.27×10^−129^ in blood, *P* = 2.386×10^−13^ in LCLs; *ULK3*, *P* = 3.17×10^−17^ in blood, *P* = 1.043×10^−20^ in LCLs, *P* = 3.21×10^−35^ in monocytes) (S1 Table) [10–13]. These eQTL associations suggest *CSK* and *ULK3* as strong candidates for a causative gene of the 15q24 locus. *CSK*, C-terminal Src kinase, down regulates the tyrosine kinase activity of Src, a proto-oncogene [14] and *Csk* is involved in vascular development since the *Csk* knockout mouse embryo fails to form normal sprouting and to remodel the vascular network [15]. Activation of Csk by angiotensin II (Ang II; a vasoconstrictor) is reduced in vascular smooth muscle cells (VSMCs) from Spontaneously Hypertensive Rats (SHR), leading to activation of Src, a target of Csk [16].

*ULK3*, serine/threonine kinase, mediates Hedgehog signaling during embryonic development and has been implicated in the maintenance of tissue homeostasis and neurogenesis in adults [17]. During the cell cycle, phosphorylation of Ulk3 is important in the G2/M phase, suggesting a role in cell cycle progression [18].

There has been no functional study to support the link of these genes to blood pressure. Based on eQTL analysis studies, mouse homologs *Csk*, *Ulk3*, and *Cyp1a2* (negative control) were examined for blood pressure change in siRNA-injected mice after testing for *in vivo* silencing by siRNAs injection. We showed that only *Csk* siRNA injection increased blood pressure while *Ulk3* and *Cyp1a2* siRNA injections did not change it. Our results along with the eQTL analysis indicate that *CSK* is a causative gene in the 15q24 locus. We also confirmed that haploinsufficiency of *Csk* increased blood pressure in *Csk*^+/-^ heterozygote mice compared to the wild-type. Further, inhibition of Src, a Csk target ameliorated hypertension in *Csk*^+/-^ heterozygote mice, suggesting that Csk regulates blood pressure through Src.

## Materials and Methods

### Animal research and ethics statement

All mice were housed and handled in a pathogen-free facility of College of Pharmacy at Kyung Hee University in compliance with the Guide for the Care and Use of Laboratory Animals, fully accredited by the Association for Assessment and Accreditation of Laboratory Animal Care. The mice were maintained on a 12-hour light/dark cycle at a constant temperature with free access to food (LabDiet 5L79, St. Louis, MO, USA) and water. Every effort was made to minimize the number of sacrificed animals and their suffering. Animals were anesthetized by intraperitoneal (i.p.) injection of tribromoethanol (18 ml of the working solution per kg body weight), of which the working solution was diluted 40-fold in 0.9% NaCl from the stock solution (10 g 2,2,2, tribromoethanol dissolved in 10 ml tertiary amyl alcohol) and euthanized by removing the heart. The experiment was approved by the local committee for the Care and Use of Laboratory Animals, College of Pharmacy Institutional Animal Care and Use Committee (license number: KHP-2010-04-06).

Female BALB/c mice (Japan SLC, Inc., Shizuoka, Japan) were used at age 7–9 weeks for experiment. *Csk* knockout heterozygote mice, B6.129S-*Csk*^*tm1Sor*^/J, were purchased from The Jackson Laboratory (Bar Harbor, ME). Heterozygote and wild-type homozygote pairs were mated, and the progeny was genotyped by PCR with the primer set using the wild-type (5’-CGCAGTCTACGAGGTGATGA-3’), the mutant (5’-CCTTCTATCGCCTTCTTGACG-3’) and the common reverse primer (5’-GGGCTCAGTTCAAGTTCAGG-3’).

### The *in vivo* delivery of siRNA

The selection and *in vivo* delivery of siRNA have been previously described [19–21]. In brief, more than 3 siRNAs per gene (*Csk*, *Ulk3*, and *Cyp1a2*) were synthesized by Genolution (Seoul, Korea), and only one of them was selected for *in vivo* injection. To determine their silencing efficacy, 20 nM of the siRNAs was transfected into B16F10 cells and NIH3T3 cells using G-Fectin (Genolution, Seoul, Korea) and Lipofectamine 2000 (Invitrogen, Carlsbad, CA) respectively according to their manufacturer’s instructions. *TRP53* siRNA was used as a positive control for all *in vitro* transfection experiments. The target siRNAs and scrambled control siRNA sequences are shown in S4 Table.

For the *in vivo* delivery into mice, polyethylenimine called as in vivo-jetPEI™ (Polyplus, 201-10G, Illkirch-Graffenstaden, France) was used as the transfection reagent. According to the manufacturer’s instruction, 50 μg of siRNA and 6.5 μl of in vivo-jetPEI (N/P charge ratio of 6) were diluted with 50 μl of 10% glucose solution and 50 μl of sterile H_2_O. The solution was vortexed gently and left for 15 min at room temperature. The mixture was injected into the tail veins of 7–9-week-old, female BALB/c mice, and 24 hours after the last injection, the treated mice were used for experiments (i.e., blood pressure measurement, collection of tissues for mRNA quantitation or for Western blotting analysis). The 1-, 2- or 3-time injection means once per day for 1, 2, or 3 days.

### Quantitative real-time PCR

Total RNA was extracted from the tissues of siRNA-injected mice 24 hours after the last injection using TRIzol (Invitrogen, Carlsbad, CA, USA). cDNA was synthesized from 500 ng of total RNA using the PrimeScript™ RT kit (TaKaRa, Shiga, Japan) according to the manufacturer’s protocol. Quantitative real-time PCR analysis was performed using SYBR Green I (TaKaRa, Shiga, Japan) on the ABI Step One Real-Time PCR system (Applied Biosystems, Foster, CA) using the following program: 45 cycles at 95°C for 10 s, 60°C for 15 s, and 72°C for 20 s. The primer sequences are shown in S5 Table.

To present the data as the fold-change of relative expression of “case” (e.g. target siRNA-treated, *Csk*^+/-^) over “control” (e.g. control siRNA-treated, *Csk*^+/+^), the 2^-∆∆Ct^ method was used as previously described [22]. First, the *Gapdh* Ct value was subtracted from each target gene Ct value to obtain the delta Ct (∆Ct) value for normalization and the ∆Ct of the “control” sample was averaged. Second, the averaged ∆Ct value of the “control” sample was subtracted from each ∆Ct value of the “case” sample to obtain the delta delta Ct (∆∆Ct = ∆Ct case—∆Ct control). Third, the ∆∆Ct values are converted to the linear form using the term 2^-∆∆Ct^. The Standard Error of the Mean (SEM) is calculated from the final 2^-∆∆Ct^ values. Statistical significance was calculated for differences between the 2^-∆Ct^ values of the “control” and those of the “case” samples.

### Culture of VSMC

The T/G HA-VSMC (vascular smooth muscle cell) line (CRL-1999) was purchased from ATCC (American Type Culture Collection, Virginia, USA). This line of VSMC is a normal human cell line established from the normal aorta of an 11-month old child and one of the best-characterized cellular models for the analysis of vascular smooth muscle cell biology. Cells were cultured in F-12K Medium (ATCC, No. 30–2004) supplemented with 0.05 mg/ml ascorbic acid, 0.01 mg/ml bovine insulin, 0.01 mg/ml human transferrin, 10 ng/ml sodium selenite, 0.03 mg/ml Endothelial Cell Growth Supplement, 10 mM HEPES, 10 mM TES, and 10% fetal bovine serum under a 5% CO_2_ in air-ventilated incubator. To knock-down *Csk*, VSMCs were transiently transfected with control or *Csk* siRNA using Lipofectamine 2000 and treated for 24 hours with DMSO or PP2 after 24 hours transfection and then proteins were extracted for Western blotting.

### Western blotting

Total proteins were extracted from mouse tissues using PRO-PREP protein extraction solution (Intron Biotechnology, Gyeonggi-Do, Korea) according to the manufacturer's instruction. Protein concentrations were measured by Bradford assay [23].

Total proteins (10 to 40 μg) were separated by 8–10% SDS-PAGE and transferred to a nitrocellulose membrane (Pall, Ann Arbor, MI, USA). The membrane was blocked in 5% skim milk for 30 min at room temperature and subsequently incubated with an antibody overnight at 4°C; anti-Actin (sc-1616, goat polyclonal, 1:1000), and anti-Csk (sc-286, rabbit polyclonal, 1:1000) from Santa Cruz Biotechnology (Santa Cruz, CA, USA); anti-phospho-Src^Y416^ (#2101, rabbit polyclonal, 1:1000), anti-phospho-Src^Y527^ (#2105, rabbit polyclonal, 1:1000), and anti-Src (#2123, rabbit polyclonal, 1:2000) from Cell Signaling Technology (Danvers, MA, USA). The blot was incubated with a horseradish peroxidase-conjugated secondary antibody (Santa Cruz Biotechnology, Inc., Santa Cruz, CA, USA) for 1 hour at room temperature. Protein signals were detected using Luminol Reagent (Santa Cruz Biotechnology, Inc., Santa Cruz, CA, USA) and exposed to x-ray films (Agfa-Health Care NV, Mortsel, Belgium).

### Blood pressure measurement

The protocol for blood pressure measurement has been previously described [19–21]. In brief, blood pressure was recorded intra-arterially with a computerized data acquisition system (AD Instruments, Bella Vista, Australia). First, mice (7-9-week old, female, 20–25 g) were anesthetized by intraperitoneal (i.p.) injection of tribromoethanol (18 ml of the working solution per kg body weight), of which the working solution was diluted 40-fold in 0.9% NaCl from the stock solution (10 g 2,2,2, tribromoethanol dissolved in 10 ml tertiary amyl alcohol). The intra-arterial catheter (a polyethylene tube; 0.2 mm I.D., 0.5 mm O.D.; Natsume, Tokyo, Japan) filled with 0.9% NaCl containing 100U/ml heparin was connected to the system. Second, while on anesthetics, a small incision was made to draw out the right carotid artery of the mouse. The catheter was inserted into the right carotid artery through a small puncture and tightly ligated with 4–0 silk suture thread around the artery. Third, blood pressure in the vessel was transmitted along the catheter to the transducer’s diaphragm (MLT0699 Disposable BP Transducer, AD Instruments, Bella Vista, Australia). The diaphragm signal was amplified through a bridge amplifier and recorded on a Power Lab system (Lab Chart 7.2, AD Instruments, Bella Vista, Australia).

Blood pressure was monitored for 2 hours after injection of the anesthetic and presented as the average value at each minute between 40 and 80 min (a linear graph) and as the average value during 40 minutes from 40 to 80 min (a bar graph) because blood pressure during the presented period was considered enough stable as evidenced by multiple repeated experiments.

To test the effect of PP2 (Merck Millipore, 529573, Darmstadt, Germany) or PP3, a negative control inhibitor (Merck Millipore, 529574, Darmstadt, Germany), blood pressure was measured in *Csk*^+/-^ and *Csk*^+/+^ mice 24 hours after i.p. injection of PP2 or PP3 at doses of 2 or 10 μg per kg body weight.

### Statistical analysis

Statistical analysis was performed using SPSS (PASW Statistics 22.0), and all data between case and control groups were analyzed by Mann-Whitney U-test since the U-test was in general considered more powerful than the t-test [24]. All data were expressed as mean ± SEM. *P* < 0.05 was considered statistically significant. * *P*<0.05, ** *P*<0.01 versus respective controls.

## Results

### Injection of *Csk*, *Ulk3*, and *Cyp1a2* siRNA reduces their respective target mRNA levels in mouse tissues

**Fig 1:**
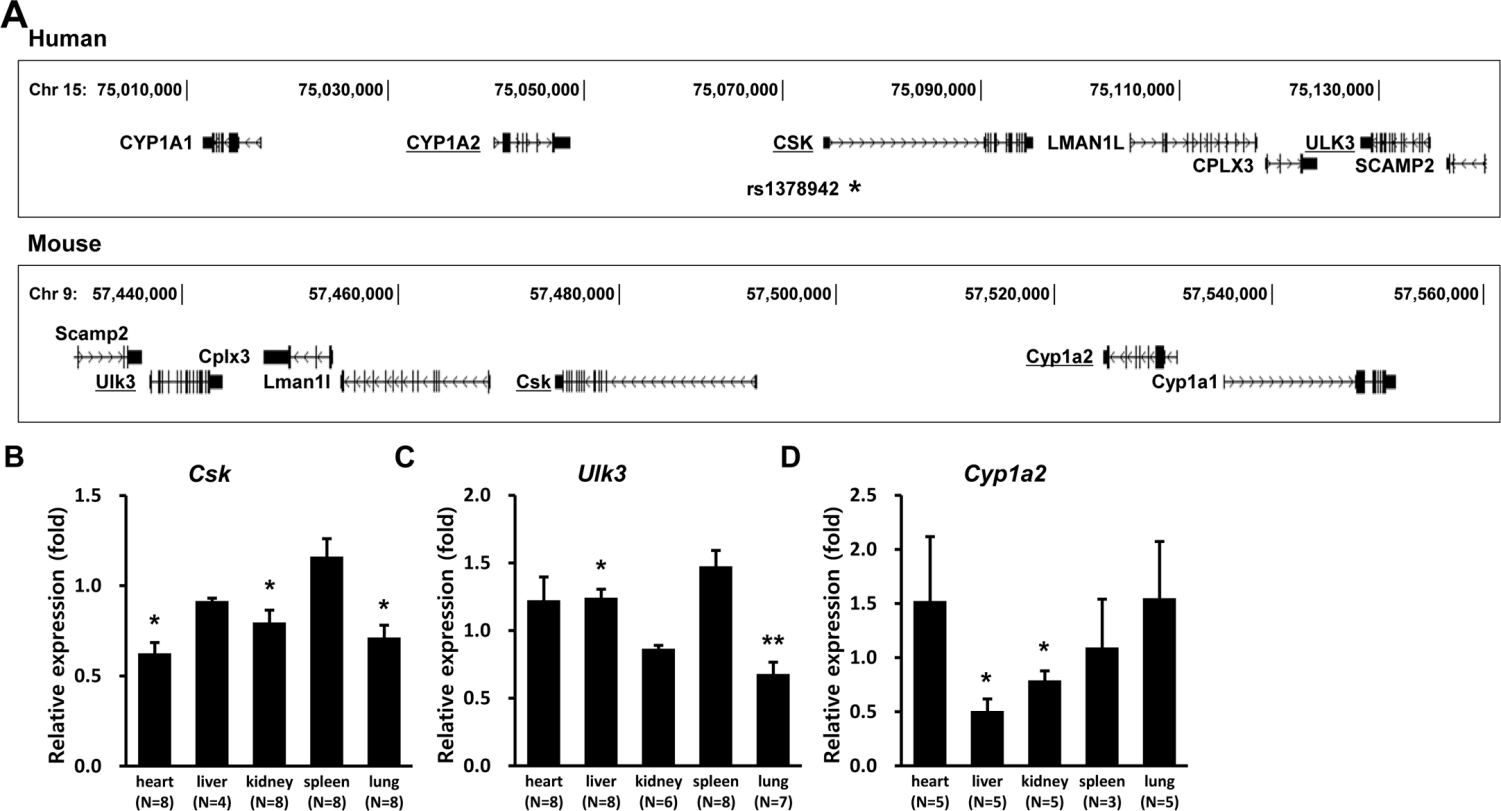
Location of candidate genes in the 15q24 GWAS locus and reduction of target gene mRNAs in siRNA-injected mice. (A) Positions of candidate genes are comparably shown on the human 15q24 locus and on the homologous mouse chromosome 9, 31.18 cM in the opposite direction; * indicates position of rs1378942, the lead SNP for blood pressure GWASs in the 15q24 locus. (B) The relative expression of candidate gene mRNAs in each tissue is presented as the fold change of relative mRNA levels in candidate gene *versus* control siRNA-injected mice after 1-time siRNA injection. Error bars show the mean ± SEM. Number in parentheses indicates the pair number of mice used for real-time PCR analysis. *P* values were calculated by Mann-Whitney U-test. * *P*<0.05 *versus* respective controls.

Fig 1A shows the locations of nearby genes including *CYP1A2*, *CSK*, and *ULK3* on the human 15q24 locus with the comparable gene locations on mouse chromosome 9 in the opposite direction. The lead SNP (rs1378942 designated as *) is located in the first intron of *CSK*. To test the effect of candidate gene variation on blood pressure, we used silencing of candidate genes by siRNA injection into mice *in vivo*. Both mouse homologs *Csk* and *Ulk3* were selected as comparably strong candidate genes among nearby genes on the 15q24 locus based on eQTL analysis studies (S1 Table). *Cyp1a2* was tested as a negative control because there has been reported neither significant association with blood pressure in GWAS or significant link to the lead SNP (rs1378942) in eQTL resources (S1 and S3 Tables). Regarding its functional role, *CYP1A2* is associated with habitual caffeine consumption, and mediates drug metabolism and the synthesis of cholesterol, steroids, and lipids (S3 Table) [25, 26].

Three siRNAs per gene were synthesized and tested for their silencing efficacies in NIH3T3 and B16F10 cells, and the most effective siRNA was selected for *in vivo* injection (S4 Table). The selected siRNAs caused *Csk*, *Ulk3*, and *Cyp1a2* levels to decline by 40.1%, 74.5%, and 44.9% in cells, respectively. The siRNAs were mixed with polyethylenimine and injected into mouse tail veins. Then, to estimate the systemic change by siRNA injection, we measured target genes mRNA levels 24 hours later by quantitative real-time PCR, comparing them between candidate gene siRNA- and control siRNA-injected mouse tissues.

After 1-time injection, *Csk* mRNA levels decreased significantly in heart by 37% (*P* = 0.038), in kidney by 20% (*P* = 0.038), and in lung by 29% (*P* = 0.017), respectively, and *Ulk3* mRNA levels fell in lung by 32% (*P* = 0.001) (Fig 1B and 1C). *Cyp1a2* mRNA levels fell in liver by 49% (*P* = 0.036) and in kidney 21% (*P* = 0.032) (Fig 1D).

### *Csk* siRNA injection increases blood pressure

To study the functional effect of siRNA injection, blood pressure was measured 24 hours after the last injection of *Csk*, *Ulk3*, or *Cyp1a2* (negative control) siRNAs.

**Fig 2:**
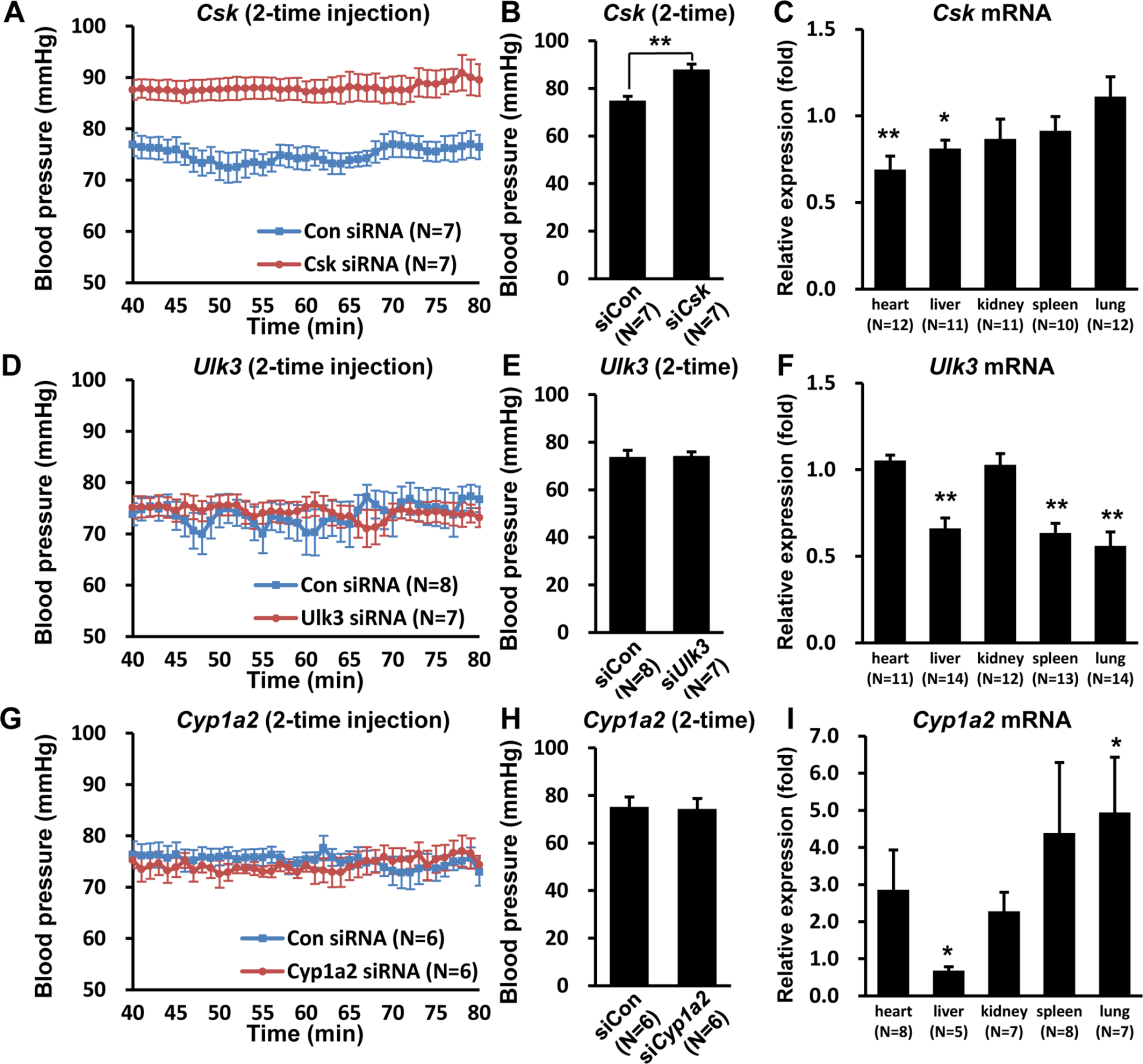
Increase in blood pressure in *Csk* siRNA-injected mice. (A, D, and G) The line graph shows blood pressure as the average value at each minute from 40 min to 80 min after anesthesia in (A) 2- time *Csk* siRNA-, (D) 2-time *Ulk3* siRNA-, and (G) 2-time *Cyp1a2* siRNA-injected mice. (B, E, and H) The bar graph shows the mean blood pressure for each group from 40 min to 80 min. (C, F, and I) Graphs show the relative expression of candidate gene mRNA in each tissue after 2-time siRNA injection. * *P*<0.05, ** *P*<0.01 *versus* respective controls.

After 2-time injection, blood pressure did not change in *Cyp1a2* siRNA-injected group (74.3 mmHg, N = 6) compared with control groups (75.2 mmHg, N = 6) (*P* = 1.000) (Fig 2G and 2H). However, *Csk* siRNA-injected mice (88.2 mmHg, N = 7) showed significantly increased blood pressure compared with control groups (74.9 mmHg, N = 7) (*P*<0.001) (Fig 2A and 2B). Interestingly *Ulk3* siRNA-injected mice (74.3 mmHg, N = 7) did not show significant changes in blood pressure *versus* control mice (73.9 mmHg, N = 8) (*P* = 1.000) (Fig 2D and 2E).

To confirm reduction of the target gene mRNA, *Csk*, *Ulk3*, and *Cyp1a2* mRNA levels were measured in mice tissues after 2-time injection of each siRNA. *Csk* mRNA levels decreased significantly in heart by 31% (*P* = 0.001) and in liver by 19% (*P* = 0.01), respectively, and *Ulk3* mRNA levels also decreased in liver by 34% (*P* = 0.001), in spleen by 37% (*P* = 0.001), and in lung by 44% (*P* = 0.003), respectively (Fig 2C and 2F). *Cyp1a2* transcripts also fell in liver by 32% (*P* = 0.045) after 2-time injection (Fig 2I). The results showed that only *Csk* gene knock-down induced a significant change in blood pressure although all three genes (*Csk*, *Ulk3*, and *Cyp1a2)* were knock-downed by siRNA injections in various tissues.

### Csk protein levels stay decreased in mouse tissues after siRNA injection

**Fig 3:**
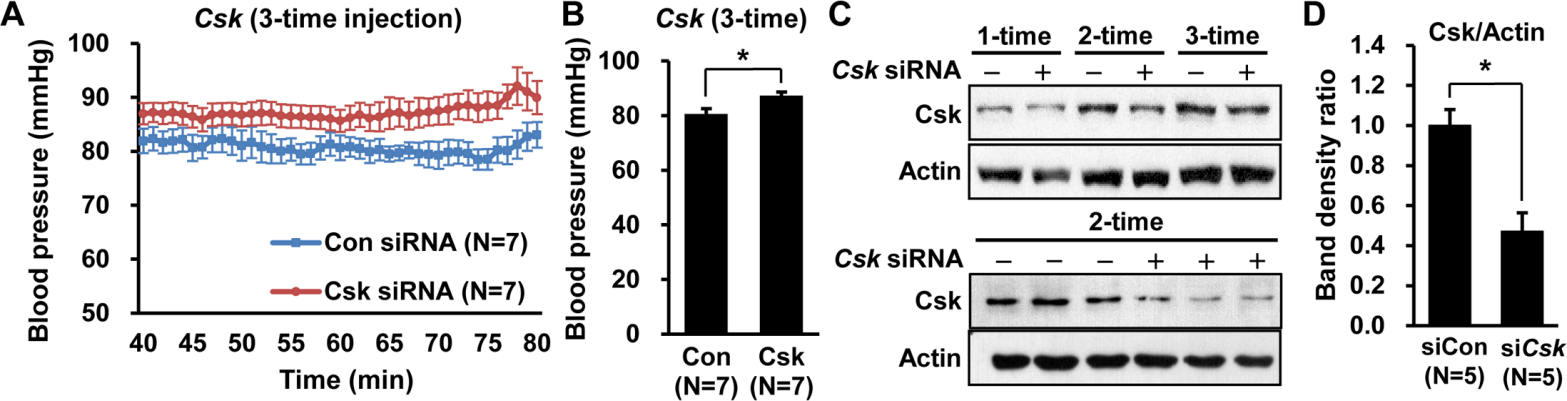
Increase in blood pressure in 3-time *Csk* siRNA-injected mice and change in protein levels in *Csk* siRNA-injected mice. (A) The line graph and (B) the bar graph show blood pressure from 40 min to 80 min after anesthesia in 3-time *Csk* siRNA-injected mice. (C) Csk protein levels in lung are shown for 1-, 2-, and 3-time siRNA injected mice following Western blotting. (D) Csk protein levels in lung are presented as the ratio of 5 independent protein band densities for 2-time siRNA injected mice.

To test the lasting effect of siRNA silencing, *Csk* siRNAs were injected into mice once per day for 3 days (3-time injection). Blood pressure significantly differed between *Csk* siRNA-treated mice (87.4 mmHg, N = 7) and control mice (80.6 mmHg, N = 7) (*P* = 0.017) even after 3-time injection (Fig 3A and 3B).

To study Csk protein levels in *Csk* siRNA-injected mice, Western blotting for Csk was performed using mouse lung tissues. Csk protein levels declined after all 1-, 2- and 3-time injections in lung tissues (Fig 3C). After 2-time injection of *Csk* siRNA, Csk protein levels fell in lung by 53% (*P* = 0.016) (Fig 3C and 3D). These data imply that Csk protein levels may stay decreased systematically after repeated injections of siRNAs despite of rebounding mRNA levels.

### Blood pressure increases in *Csk* heterozygote mice

**Fig 4:**
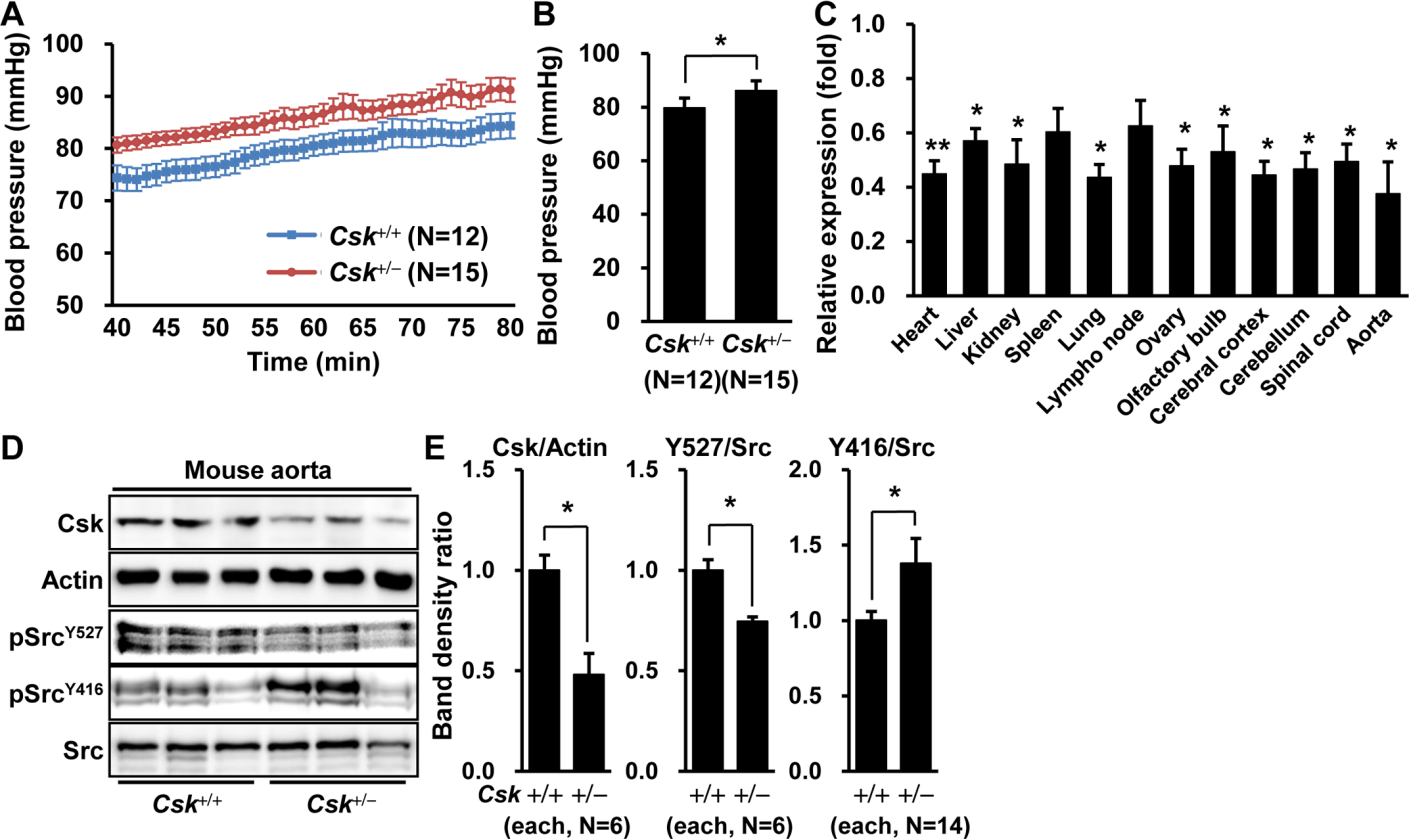
Increase in blood pressure in *Csk*^+/-^ mice. (A, and B) Blood pressure is expressed as (A) the average value at each minute and (B) the mean blood pressure for each group from 40 min to 80 min after anesthesia in *Csk*^+/-^ and the wild-type. (C) The relative expression of *Csk* mRNA in each tissue is expressed as the fold change of relative levels in *Csk*^+/-^ (N = 7) *versus Csk*^+/+^ (N = 5), normalized by *Gapdh*. (D) The representative blots of Csk and phosphorylated Src protein levels are shown following Western blotting in aorta of *Csk*^+/-^ and *Csk*^+/+^ mice. (E) Protein levels in aorta are presented as the ratio of band densities for *Csk*^+/-^ mice to those for *Csk*^+/-^. Number in parentheses indicates the number of mice used for the Western blotting.

To confirm the blood pressure change in *Csk* siRNA-injected mice, we examined *Csk* knockout mice. Because the *Csk* knockout homozygote (*Csk*^-/-^) was embryonic-lethal, the *Csk* heterozygote (*Csk*^+/-^) mice were compared with the wild-type (*Csk*^+/+^) mice. The *Csk* mRNA level in *Csk* heterozygotes decreased in most tissues by half (Fig 4C). Consistent with the results in *Csk* siRNA-injected mice, blood pressure in *Csk*^+/-^ mice was significantly higher than in the wild-type (*Csk*^+/-^, 86 mmHg, N = 12; *Csk*^+/+^, 80 mmHg, N = 15; *P* = 0.021) (Fig 4A and 4B).

Since Csk is a negative regulator of Src protein [14] and Src plays a role in contraction of vascular smooth muscle cells [27], Western blotting analysis was performed for Csk and phosphorylated Src proteins in the aorta of *Csk*^+/-^ and *Csk*^+/+^ mice. Csk protein levels were reduced by 52% (*P* = 0.002) in the *Csk*^+/-^ aorta (Fig 4D and 4E). While the total Src protein was not changed in *Csk*^+/-^ aorta, phosphorylation of Src^Y416^ (active) significantly increased by 38% (*P* = 0.046) and that of Src^Y527^ (inactive) decreased by 25% (*P* = 0.01), suggesting Src as a target of Csk in the aorta (Fig 4D and 4E).

### Inhibition of Src decreases high blood pressure in *Csk* heterozygote mice

**Fig 5:**
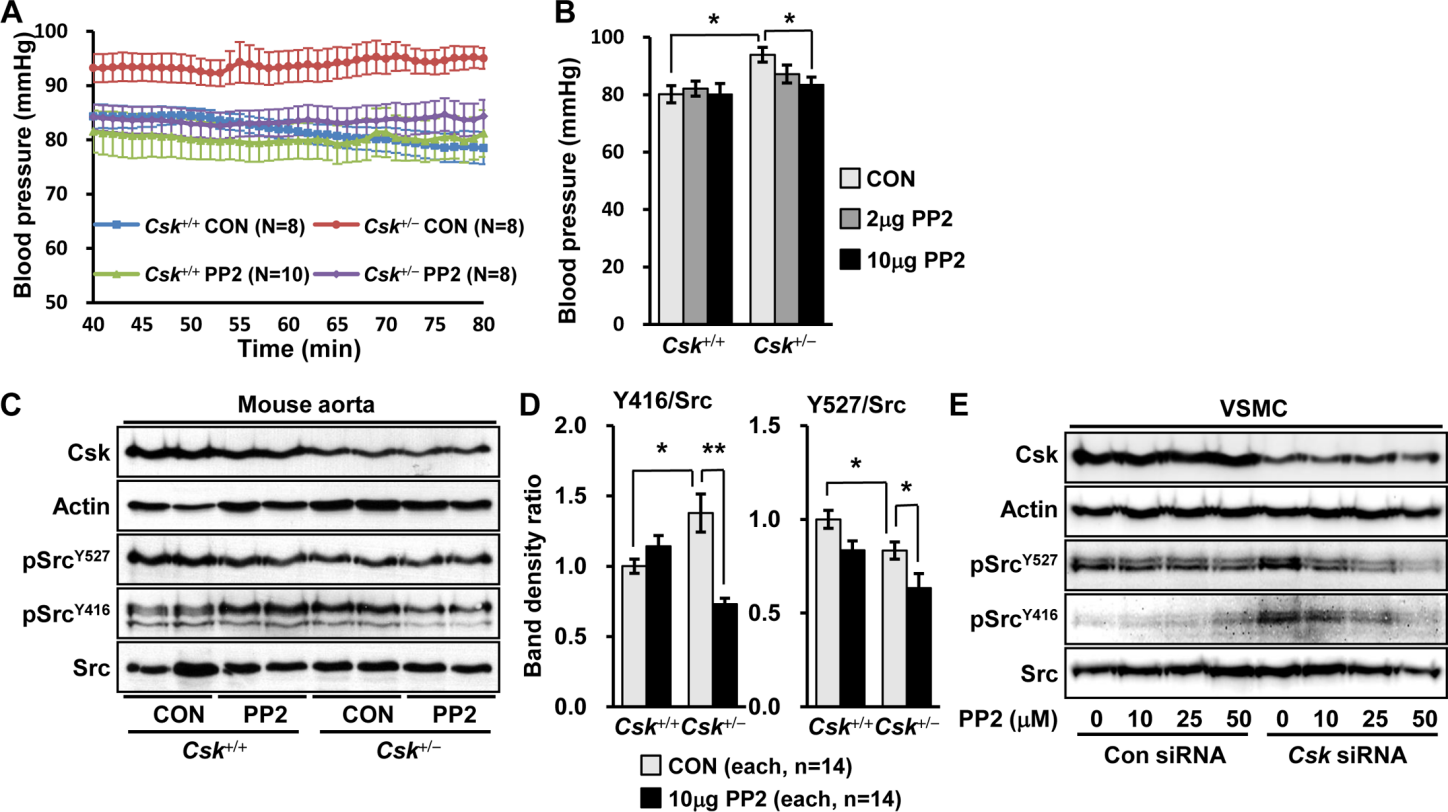
Decrease in blood pressure by PP2, a Src inhibitor in *Csk*^+/-^ mice. (A) *Csk*^+/-^ and *Csk*^+/+^ mice were i.p. injected with DMSO as control (CON) or PP2 (10 μg per kg body weight), a Src inhibitor, and blood pressure was measured 24 hours after injections. Blood pressure is expressed as the average value at each minute from 40 min to 80 min after anesthesia. (B) Blood pressure was measured in *Csk*^+/-^ and *Csk*^+/+^ mice treated with DMSO or PP2 (2, and 10 μg per kg body weight) and the data is shown as the mean blood pressure for each group from 40 min to 80 min. (C) The representative blots of Csk and Src protein are shown following Western blotting in aorta of *Csk*^+/-^ and *Csk*^+/+^ mice treated with DMSO or PP2 (10 μg per kg body weight). (D) Protein levels are presented as the ratio of protein band densities in *Csk*^+/-^ aorta to the wild-type. (E) Human VSMC cells were transfected by *Csk* siRNA and then treated with DMSO or PP2 (10, 25, and 50 μM). Phosphorylated Src and Csk protein levels are shown following Western blotting.

To study whether Csk involves Src in modulating blood pressure, we examined the effect of PP2, a Src inhibitor in *Csk*^+/-^ mice. High blood pressure in *Csk*^+/-^ mice (93.9 mmHg, N = 8) was significantly reduced 24 hours after i.p. injection of 10 μg PP2 per kg body weight (83.5 mmHg, N = 8; *P* = 0.038) (5A and 5B). Lesser dose of PP2 seemed to decrease blood pressure without significance in *Csk*^+/-^ mice (2 μg/kg PP2, 87.2 mmHg, N = 5, *P* = 0.284) while PP2 did not affect the basal blood pressure in the wild-type (0μg/kg, 80.2 mmHg, N = 8; 2 μg/kg, 82.1 mmHg, N = 8; 10 μg/kg, 80.2 mmHg, N = 10) (Fig 5B). We studied the effect of PP3, a negative control inhibitor of PP2 and did not see any significant change of blood pressure by its treatment (*Csk*^+/+^, 80 mmHg, N = 18; *Csk*^+/-^, 89 mmHg, N = 8; *Csk*^+/-^ + 10 μg/kg PP3, 89 mmHg, N = 5), indicating that PP2 was specific in its effect on blood pressure (S1 Fig).

To confirm inhibition of Src by PP2, we performed Western blotting analysis for phosphorylated Src proteins in the aorta of *Csk*^+/-^ and *Csk*^+/+^ mice treated with 10 μg/kg PP2. As shown in Fig 5C and 5D, phosphorylated Src^Y416^ (active) was significantly reduced in the PP2-treated *Csk*^+/-^ aorta compared to vehicle-treated aorta and phosphorylated Src^Y527^ (inactive) was also slightly decreased.

Further to study regulation of Src by Csk in functionally relevant cell types, we cultured human vascular smooth muscle cells (T/G HA-VSMC, ATCC), transfected cells with *Csk* siRNA, and then treated with 10, 25, 50 μM PP2 for 24 hours. The Western blotting analysis showed that phosphorylation of Src^Y416^ was conspicuously increased by *Csk* siRNAs transfection in VSMCs consistent with the result from the *Csk*^+/-^ mice aorta (Fig 5E). Moreover, PP2 treatment dose-dependently decreased phosphorylation of Src^Y416^ in *Csk* siRNA-transfected cells (Fig 5E). Phosphorylation of Src^Y527^ did not significantly change by *Csk* siRNAs transfection, but seemed to decrease by PP2 treatment in VSMCs (Fig 5E).

These results suggest that Src is specifically involved in blood pressure regulation by Csk.

## Discussion

It has been a challenging task to identify a causative gene in a locus that is associated with blood pressure. Therefore, there are very few genes identified as causative so far despite of multiple candidates associated with blood pressure GWASs. For that reason, we utilized two-step approaches. First, we selected the most relevant candidate genes in a locus by using the eQTL analysis studies. Then, we tested the effect of gene knock-down on blood pressure after the *in vivo* delivery of candidate gene siRNA into mice.

We previously reported *ATP2B1* in the 12q21 locus [20] as a causative gene by testing the effect of target gene siRNA silencing on blood pressure. The recent eQTL study showed that the expression of *ATP2B1* transcripts was associated with rs1401982 and rs17249754 (rs1401982, *P* = 1.4×10^−24^ for all common SNPs; rs17249754, *P* = 5.5×10^−4^ for 1,167 trait-associated SNPs in blood) [11], supporting our previous study using *in vivo* siRNA silencing. The *SORT1* gene is another good example that a specific gene is identified as associated with a variant (rs599839) in the 1p13 locus by an eQTL study (*P* = 6.11×10^−55^ in liver) and that rs629301 in LD with rs599839 is associated with plasma low-density lipoprotein cholesterol (LDL-C) in GWAS (*P* = 1×10^−170^) [28, 29]. Musunuru et al. (2010) has clearly demonstrated that rs12740374 in LD with rs599839 and rs629301 is a causative variant because its polymorphism alters hepatic expression of the *SORT1* gene and knockdown by *Sort1* siRNA in mouse liver changes plasma LDL-C levels [30].

In the present study we used the eQTL resources more aggressively to sort out prominent candidates. Through mining the eQTL resources, *CSK* and *ULK3* were selected in the 15q24 locus for further study. Target gene silencing was successfully tested *in vivo* according to the protocol we have previously established. As a result, *Csk* in the 15q24 locus was identified as a causative gene for the blood pressure change and furthermore confirmed in a knockout mouse model. Therefore, the present study and previous ones support that *in vivo* siRNA silencing combined with the eQTL analysis is a very useful tool to identify a causative gene among candidate genes.

However, we observed a difference in the directionality of the effect of suppression of *Csk* in mice from the results obtained in human GWAS and eQTL studies that the ‘C’ allele of the lead SNP (rs1378942) is associated with higher blood pressure and higher *CSK* transcripts [2, 5–7, 11, 12]. We considered three possible reasons for the conflicting results. One possibility is a molecular and functional difference between the human *CSK* and mouse *Csk* gene. But it is very unlikely since the human CSK and mouse Csk proteins are identical except one residue at 123 within SH2 domain, not within a functional domain such as the kinase domain. The other one is a recording error for SNP alleles in eQTL databases. We personally corresponded with authors of Fehrmann et al. (2011) and Grundberg et al. (2012) papers but were provided with a solid confirmation for their databases. The third reason might be the lack of the functional tissue in eQTL resources. We speculate that the correct eQTL data may be unavailable until the functional tissue of *CSK* for blood pressure regulation is revealed. Despite of still unsolved conflicts, we believe that our results provide an important clue for how the decreased *Csk* gene activity is associated with hypertension in Spontaneously Hypertensive Rats (SHR) [16], as explained in the following section.

### Csk regulates blood pressure through Src

**Fig 6:**
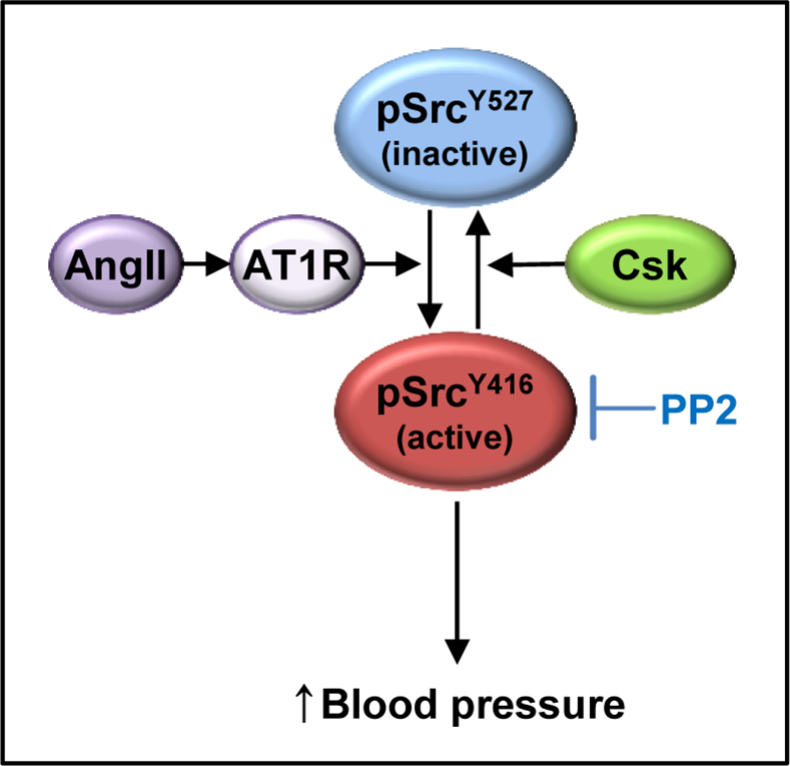
The working model of blood pressure regulation by Csk and Angiotensin II through Src. Angiotensin II (Ang II) increases blood pressure through angiotensin type 1 receptor (AT1R) dependent on the Csk/Src signal. Csk, a negative regulator of Src increases phosphorylation of Src^Y527^ (inactive) and decreases that of Src^Y416^ (active).

Csk was identified as a tyrosine kinase of Src, a non-receptor tyrosine kinase [14]. Csk inactivates Src by increasing phosphorylation of Src^Y527^, acting as a negative regulator of Src (Fig 6) [31]. Phosphorylation of Src^Y416^ is increased in the *Csk*^+/-^ mouse aorta and in *Csk* knock-down cells than in the wild-type (Fig 5C, 5D and 5E). Furthermore, inhibition of Src decreases high blood pressure in *Csk*^+/-^ mice (Fig 5A and 5B), strongly suggesting that hypertension occurs through Src.

Src is one member of eight Src family tyrosine kinases (c-Src, c-Yes, Fyn, c-Fgr, Lyn, Hck, Lck, and Blk) [31]. Among them, the activity of Src is important in Ang II-induced vasoconstriction of vascular smooth muscle cells (VSMCs). Ang II induces phosphorylation of Src^Y416^ *via* angiotensin type 1 receptor (AT1R) in rat VSMCs [32]. Ang II-induced intracellular Ca^2+^ mobilization is blunted in *Src*^-/-^ VSMC and vascular contraction is inhibited by PP2, a Src inhibitor [27]. Interestingly low activation of Csk is associated with increased Ang II-mediated Src signaling in VSMCs of Spontaneously Hypertensive Rats (SHR) [16]. These reports and our findings suggest that the Csk-Src signal may be a key regulatory point in Ang II-mediated vasoconstriction and blood pressure regulation (Fig 6).

Downstream of the Csk-Src signal, extracellular signal-regulated kinases (ERK1/2) may play an important role in vasoconstriction. In SHRs, inhibition of ERK signaling by U0126 reduces hypertension, vascular thickening, and smooth muscle myosin light chain kinase expression [33]. Moreover, Ang II-mediated ERK1/2 activation requires Src in rat VSMCs [16, 34]. Therefore, we speculate that Csk negatively modulates Ang II-mediated Src/ERK signaling in vasoconstriction of VSMCs.

### Src plays important roles in Ang II-mediated signaling in multiple tissues

Ang II is a major effector hormone of the renin-angiotensin system and has various effects that are mediated by AT1R to raise blood pressure, including direct vasoconstriction in the vascular smooth muscle, indirect vasoconstriction by noradrenaline release from the sympathetic nerve system, sodium reabsorption in the kidney, sodium and fluid retention by aldosterone secretion from the adrenal gland, and vascular growth stimulation [35].

In addition to already mentioned role of Src in VSMCs, Src activation is required for Ang II-mediated functions in the kidney and in the brain. In the renal proximal tubule-like cells, PP2 and *Src* siRNA inhibits Ang II-induced epithelial-to-mesenchymal transition (EMT) associated with progressive kidney damage [36]. Also Src is activated in Ang II-induced fluid reabsorption *via* apical Na^+^/H^+^ exchanger (NHE) and basolateral Na^+^-HCO_3_^-^ cotransporter (NBC) and *Csk* overexpression inhibits activation of both NHE and NBC [37, 38]. In the distal tubule of kidney, Src is required for inhibition of the renal outer medullary potassium (ROMK) channel during hypovolemia (low blood volume) by low potassium diet [39–42]. Therefore, hypertension by low potassium diet may be associated with the inhibitory effect of Src on the ROMK channel.

In the hypothalamus of hypertensive rats induced by two-kidney one-clip (2K1C), Src activation is higher than sham-operated rats and is more increased by Ang II [43]. Two specific Src inhibitors (PP2, SU6656) abolish increase in sympathetic nerve activity and blood pressure of 2K1C rats, suggesting that Src in the brain mediates sympathetic activation by renovascular hypertension.

Considering that Src plays regulatory roles in diverse tissues, hypertension in *Csk*^+/-^ mice may rather be the combined result of differential effects by chronic Src activation in multiple tissues (e.g., vasculature, kidney, or/and brain) than the result of a defect in one particular tissue. Therefore, to fully understand the mechanism of hypertension in *Csk*^+/-^ mice, changes in Src-associated signaling need to be further studied in each tissue.

In summary, we have identified *CSK* a causative gene in the blood pressure GWAS locus 15q24 by selecting candidate genes using the eQTL analysis studies, by verifying the functional effect of genes using *in vivo* siRNA delivery in mice, and by confirming our findings in knockout heterozygote mice. Furthermore, we showed that Csk involves Src by negatively regulating its activity to modulate blood pressure. Our result that Csk regulates blood pressure through Src suggests a novel pathway for the development of hypertension and provides a therapeutic target for the treatment of high blood pressure.

## Supporting Information

S1 Fig No effect on blood pressure by PP3 in *Csk*^+/+^ and *Csk*^+/-^ mice. (A and B) *Csk*^+/-^ and *Csk*^+/+^ mice were i.p. injected with DMSO as control (CON) or PP3 (10 μg per kg body weight), a negative control for PP2, and blood pressure was measured 24 hours after injections.
(PDF)

S1 Table eQTL Analysis. (PDF)

S2 Table The lead SNP (rs1378942) and variants with r^2^ > = 0.2 (European population) (HaploReg v3). (PDF)

S3 Table The catalog of Published Genome-Wide Association Studies for variants near the lead SNP (rs1378942, ± 1Mb boundary) (National Human Genome Research Institute). (PDF)

S4 Table Reduction of *Csk*, *Ulk3*, and *Cyp1a2* mRNA levels in cells after treatment with siRNAs. (PDF)

S5 Table Primers used for quantitative real-time PCR. (PDF)
