# Supplementary material for: Gene Silencing and Haploinsufficiency of Csk Increase Blood Pressure
Source: PLoS One. 2016 Jan 11;11(1):e0146841. doi: 10.1371/journal.pone.0146841 (PMC4713444; doi:10.1371/journal.pone.0146841)
Supplement: S1 Fig — (A and B) Csk+/- and Csk+/+ mice were i.p. injected with DMSO as control (CON) or PP3 (10 μg per kg body weight), a negative control for PP2, and blood pressure was measured 24 hours after injections. (PDF) [file pone.0146841.s001.pdf]

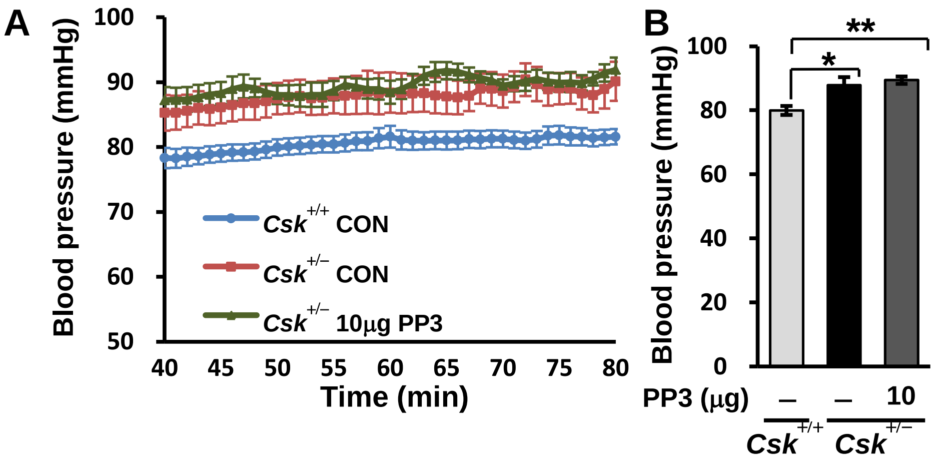

S1 Fig. No effect on blood pressure by PP3 in *Csk*$^{+/+}$ and *Csk*$^{+/-}$ mice. (A and B) *Csk*$^{+/-}$ and *Csk*$^{+/+}$ mice were i.p. injected with DMSO as control (CON) or PP3 (10 μg per kg body weight), a negative control for PP2, and blood pressure was measured 24 hours after injections.
