# Supplementary material for: Gene Silencing and Haploinsufficiency of Csk Increase Blood Pressure
Source: PLoS One. 2016 Jan 11;11(1):e0146841. doi: 10.1371/journal.pone.0146841 (PMC4713444; doi:10.1371/journal.pone.0146841)
Supplement: S1 Table — (PDF) [file pone.0146841.s002.pdf]

Supporting Information

S1 Table. eQTL Analysis

SNP ID	SNP Chr	SNP Type	Allele Assessed	Gene Symbol	P-Value	Z-score	Beta size	Tissue	Reference
rs2472299*	15	G/A	A	**CSK**	**3.55E-34**	12.19		Blood	Fehrmann et al, 2011 [1]
rs2472299*	15	G/A	A	ULK3	6.55E-18	8.62		Blood	
**rs1378942***	15	A/C	C	**CSK**	**1.97E-45**	14.15		Blood	
**rs1378942***	15	A/C	C	ULK3	3.17E-17	8.44		Blood	
rs2301249	15	G/A	A	**CSK**	**4.77E-39**	13.07		Blood	
rs2301249	15	G/A	A	ULK3	5.97E-23	9.86		Blood	
rs6495122*	15	A/C	C	**CSK**	**1.04E-25**	-10.48		Blood	
rs6495122*	15	A/C	C	ULK3	1.51E-10	-6.40		Blood	
rs6495122*	15	A/C	C	SCAMP2	4.51E-10	6.24		Blood	
rs2472299*	15	G/A	G	CYP1A2	6.73E-02		-0.01027	LCL	Grundberg et al, 2012 [2]
rs2472299*	15	G/A	G	CSK	4.25E-10		-0.07390	LCL	
rs2472299*	15	G/A	G	LMAN1L	7.63E-01		-0.00247	LCL	
rs2472299*	15	G/A	G	CPLX3	1.83E-01		0.00740	LCL	
rs2472299*	15	G/A	G	**ULK3**	**3.06E-22**		-0.11280	LCL	
rs2472299*	15	G/A	G	SCAMP2	2.11E-01		0.02001	LCL	
**rs1378942***	15	A/C	C	CYP1A2	1.64E-01		0.00757	LCL	
**rs1378942***	15	A/C	C	CSK	2.39E-13		0.08420	LCL	
**rs1378942***	15	A/C	C	LMAN1L	8.09E-01		-0.00191	LCL	
**rs1378942***	15	A/C	C	CPLX3	4.71E-01		-0.00388	LCL	
**rs1378942***	15	A/C	C	**ULK3**	**1.04E-20**		0.10530	LCL	
**rs1378942***	15	A/C	C	SCAMP2	1.67E-02		-0.03717	LCL	
rs2301249	15	C/T	T	CYP1A2	2.11E-01		0.00710	LCL	
rs2301249	15	C/T	T	CSK	3.55E-11		0.07920	LCL	
rs2301249	15	C/T	T	LMAN1L	4.46E-01		0.00632	LCL	
rs2301249	15	C/T	T	CPLX3	2.01E-01		-0.00717	LCL	
rs2301249	15	C/T	T	**ULK3**	**2.93E-24**		0.11950	LCL	
rs2301249	15	C/T	T	SCAMP2	4.24E-02		-0.03283	LCL	
rs7085	15	T/C	T	CYP1A2	2.07E-01		0.00718	LCL	
rs7085	15	T/C	T	CSK	3.80E-11		0.07920	LCL	
rs7085	15	T/C	T	LMAN1L	4.48E-01		0.00632	LCL	
rs7085	15	T/C	T	CPLX3	2.03E-01		-0.00715	LCL	
rs7085	15	T/C	T	**ULK3**	**3.06E-24**		0.11970	LCL	
rs7085	15	T/C	T	SCAMP2	4.04E-02		-0.03323	LCL	
rs6495122*	15	A/C	C	CYP1A2	1.39E-01		-0.00772	LCL	
rs6495122*	15	A/C	C	CSK	1.16E-10		-0.07100	LCL	
rs6495122*	15	A/C	C	LMAN1L	7.49E-01		0.00244	LCL	
rs6495122*	15	A/C	C	CPLX3	5.88E-01		0.00280	LCL	
rs6495122*	15	A/C	C	**ULK3**	**6.78E-17**		-0.09020	LCL	
rs6495122*	15	A/C	C	SCAMP2	6.55E-04		0.05077	LCL	
rs2472299*	15	A/G		**ULK3**	**2.16E-38**			Monocyte	Zeller et al,

**rs1378942***	15	C/A		**ULK3**	**3.21E-35**		Monocyte	2010 [3]
**rs1378942***	15	A/C	C	**CSK**	**1.27E-129**	24.22	Blood	Westra et al, 2013 [4]
**rs1378942***	15	A/C	C	ULK3	5.71E-33	11.96	Blood	
**rs1378942***	15	A/C	C	SCAMP2	3.27E-06	-4.65	Blood	
rs7085	15	C/T	T	**CSK**	**1.55E-131**	24.40	Blood	
rs7085	15	C/T	T	ULK3	2.48E-48	14.61	Blood	
rs6495122*	15	C/A	C	**CSK**	**1.56E-79**	-18.88	Blood	
rs6495122*	15	C/A	C	ULK3	3.44E-23	-9.92	Blood	
rs6495122*	15	C/A	C	SCAMP2	7.52E-25	10.29	Blood	

*These SNPs have been previously reported and cataloged by National Human Genome Research Institute as in S3 Table.

None of SNPs that are in LD ($r^2 > 0.80$) with rs1378942 has been reported for eQTL (S2 Table).

**Study**	Fehrmann et al, 2011
**Population**	United Kingdom, Netherlands / N=1,469
**Genotype**	NCBI36.3 / Illumina HumanHap300 Quad platform / chr1-23 (MAF > 5%; call-rate > 95%)
**Expression / Tissue**	Illumina HumanRef-8 v2, HT-12 v3 / Peripheral blood
**eQTL Analysis on 289,044 common SNPs**	Spearman's correlation P-value threshold: < 1.73E-3 for *cis*-eQTLs (FDR < 0.05) / 250 Kb for *cis*-radius
**eQTL Analysis on 1,167 trait-associated SNPs**	Spearman's correlation P-value threshold: < 3.7E-3 for *cis*-eQTLs (FDR < 0.05) / 250 Kb for *cis*-radius

MAF, minor allele frequency; FDR, false discovery rate

**Study**	Grundberg et al, 2012 / MuTHER Resource / www.muther.ac.uk
**Population**	United Kingdom / N=837
**Genotype**	NCBI36 / HapMap2 Imputation / chr1-23 (MAF > 5%; INFO > 0.8)
**Expression / Tissue**	Illumina HumanHT-12 v3 / Lymphoblastoid cell line (LCL)
**eQTL Analysis**	MuTHER format (FDR < 0.01) / 1 Mb for *cis*-radius

**Study**	Zeller et al, 2010
**Population**	Germany, Gutenberg Heart Study (GHS) / N=1,490
**Genotype**	NCBI36.3 / Affymetrix 6.0 array / chr1-23 (MAF > 1%, call rate > 98%, FDR > 1%)
**Expression / Tissue**	Illumina Human HT-12 v3 / Monocytes
**eQTL Analysis**	P-value threshold: < 5.78E-12 for eQTL / 1 Mb for *cis*-radius

**Study**	Westra et al, 2013
**Population**	European, 7 cohorts / N=5,311
**Genotype**	NCBI36.3 / multiple genotyping platforms & Imputation / chr1-23 (MAF > 5%, Hardy-Weinberg equilibrium P value >0.001)
**Expression / Tissue**	Illumina Human HT-12v3, HT12v4, H8v2 / Peripheral blood
**eQTL Analysis**	FDR < 0.05 for eQTL / 250 Kb for *cis*-radius

1. Fehrmann RS, Jansen RC, Veldink JH, Westra HJ, Arends D, Bonder MJ, *et al.* Trans-eqtls reveal that independent genetic variants associated with a complex phenotype converge on intermediate genes, with a major role for the hla. *PLoS genetics*. 2011;7:e1002197

2. Grundberg E, Small KS, Hedman AK, Nica AC, Buil A, Keildson S, *et al.* Mapping cis- and trans-regulatory effects across multiple tissues in twins. *Nature genetics*. 2012;44:1084-1089
3. Zeller T, Wild P, Szymczak S, Rotival M, Schillert A, Castagne R, *et al.* Genetics and beyond--the transcriptome of human monocytes and disease susceptibility. *PloS one*. 2010;5:e10693
4. Westra HJ, Peters MJ, Esko T, Yaghootkar H, Schurmann C, Kettunen J, *et al.* Systematic identification of trans eqtls as putative drivers of known disease associations. *Nature genetics*. 2013;45:1238-1243
