# Supplementary material for: Gene Silencing and Haploinsufficiency of Csk Increase Blood Pressure
Source: PLoS One. 2016 Jan 11;11(1):e0146841. doi: 10.1371/journal.pone.0146841 (PMC4713444; doi:10.1371/journal.pone.0146841)
Supplement: S2 Table — (PDF) [file pone.0146841.s003.pdf]

S2 Table. The lead SNP (rs1378942) and variants with r^2 >= 0.2 (European population) (HaploReg v3).

Red shadow, the lead SNP (rs1378942).

Yellow shadow, SNPs whose eQTL data are available.

Green shadow, SNPs that are in linkage disequilibrium (LD, r^2 > 0.80) with rs1378942.

pos (hg19)	LD (r^2)	LD (D')	variant	Ref	Alt	AFR freq	AMR freq	ASN freq	EUR freq	GENCODE genes	dbSNP func annot
chr15:75031521	0.49	0.82	rs2472298	G	A	0.51	0.74	0.62	0.68	9.7kb 5' of CYP1A2	
chr15:75033400	0.5	0.84	rs2472299	A	G	0.54	0.74	0.63	0.69	7.8kb 5' of CYP1A2	
chr15:75033909	0.5	0.84	rs2472300	A	G	0.54	0.74	0.63	0.69	7.3kb 5' of CYP1A2	
chr15:75035256	0.49	0.83	rs2445618	T	C	0.54	0.74	0.63	0.68	5.9kb 5' of CYP1A2	
chr15:75035503	0.37	0.66	rs55868854	A	C	0.53	0.72	0.61	0.65	5.7kb 5' of CYP1A2	
chr15:75035612	0.46	0.82	rs113294190	A	G	0.18	0.36	0.17	0.52	5.6kb 5' of CYP1A2	
chr15:75041917	0.5	0.85	rs762551	C	A	0.54	0.74	0.63	0.69	CYP1A2	intronic
chr15:75044238	0.81	0.93	rs2472304	G	A	0.04	0.38	0.16	0.59	CYP1A2	intronic
chr15:75047426	0.81	0.93	rs2470890	C	T	0.03	0.38	0.16	0.59	CYP1A2	synonymous
chr15:75049943	0.79	0.93	rs2960192	G	A	0.05	0.38	0.16	0.58	1.4kb 3' of CYP1A2	
chr15:75050854	0.51	0.85	rs12591078	T	C	0.53	0.74	0.62	0.69	2.3kb 3' of CYP1A2	
chr15:75052495	0.78	0.94	rs12903896	C	T	0.04	0.38	0.16	0.58	4kb 3' of CYP1A2	
chr15:75052771	0.54	0.83	rs11854147	T	C	0.2	0.46	0.39	0.66	4.2kb 3' of CYP1A2	
chr15:75052820	0.49	0.85	rs11072502	G	A	0.54	0.74	0.62	0.69	4.3kb 3' of CYP1A2	
chr15:75052911	0.5	0.85	rs11072504	T	C	0.53	0.74	0.62	0.69	4.4kb 3' of CYP1A2	
chr15:75052927	0.47	0.93	rs11857376	A	G	0.03	0.29	0	0.46	4.4kb 3' of CYP1A2	
chr15:75052935	0.49	0.83	rs11072505	A	G	0.53	0.74	0.62	0.68	4.4kb 3' of CYP1A2	
chr15:75052994	0.56	0.85	rs11072506	A	G	0.43	0.71	0.55	0.66	4.5kb 3' of CYP1A2	
chr15:75054866	0.8	0.92	rs11072507	G	C	0.04	0.38	0.19	0.59	6.3kb 3' of CYP1A2	
chr15:75057203	0.52	0.89	rs4886406	G	T	0.56	0.75	0.62	0.7	8.7kb 3' of CYP1A2	
chr15:75057747	0.75	0.95	rs201618682	AT	A	0.05	0.37	0.19	0.56	9.2kb 3' of CYP1A2	
chr15:75059627	0.79	0.93	rs12903541	A	G	0.08	0.4	0.2	0.58	11kb 3' of CYP1A2	
chr15:75061916	0.86	0.96	rs11632414	A	G	0.04	0.38	0.18	0.59	12kb 5' of CSK	
chr15:75061929	0.86	0.96	rs12909307	A	G	0.04	0.38	0.18	0.59	12kb 5' of CSK	
chr15:75062397	0.88	0.96	rs11072508	C	T	0.04	0.38	0.18	0.59	12kb 5' of CSK	
chr15:75063573	0.54	0.9	rs1543927	T	C	0.55	0.75	0.62	0.7	11kb 5' of CSK	
chr15:75065644	1	1	rs4886410	G	C	0.04	0.39	0.18	0.61	8.8kb 5' of CSK	
chr15:75069282	0.64	1	rs936226	C	T	0.53	0.76	0.62	0.71	5.1kb 5' of CSK	
chr15:75070196	1	1	rs12905199	A	G	0.03	0.39	0.18	0.61	4.2kb 5' of CSK	
chr15:75072558	0.65	0.99	rs4886629	G	C	0.47	0.55	0.57	0.7	1.8kb 5' of CSK	
chr15:75077367	1	1	rs1378942	C	A	0.03	0.39	0.18	0.61	CSK	intronic
chr15:75078343	0.37	0.94	rs56228306	C	T	0.07	0.25	0.18	0.39	CSK	intronic
chr15:75079474	0.32	-1	rs34933034	G	A	0.02	0.14	0.03	0.17	CSK	intronic
chr15:75080150	1	1	rs1378941	C	A	0.03	0.39	0.18	0.61	CSK	intronic
chr15:75080685	0.66	1	rs8033381	G	A	0.53	0.55	0.58	0.7	CSK	intronic
chr15:75080872	0.66	1	rs2168519	T	C	0.47	0.55	0.58	0.7	CSK	intronic
chr15:75081078	1	1	rs2168518	G	A	0.03	0.39	0.18	0.61	MIR4513	intronic
chr15:75081745	0.66	1	rs3784790	G	C	0.54	0.55	0.57	0.7	CSK	intronic
chr15:75082552	1	1	rs3784789	C	G	0.03	0.39	0.18	0.61	CSK	intronic
chr15:75083494	1	1	rs1378940	C	A	0.03	0.39	0.18	0.61	CSK	intronic
chr15:75083912	0.66	1	rs12442901	A	G	0.56	0.56	0.58	0.7	CSK	intronic
chr15:75084281	0.68	1	rs1350193	G	C	0.56	0.56	0.58	0.69	CSK	intronic
chr15:75086386	0.65	0.99	rs72730503	G	A	0.47	0.55	0.57	0.7	CSK	intronic
chr15:75086504	0.51	0.75	rs62006565	T	C	0.45	0.5	0.51	0.58	CSK	intronic
chr15:75086534	0.72	0.96	rs62006566	C	T	0.15	0.38	0.2	0.54	CSK	intronic
chr15:75086545	0.68	0.96	rs62006567	C	T	0.03	0.36	0.16	0.53	CSK	intronic
chr15:75086589	0.52	0.95	rs35213055	C	T	0.08	0.33	0.15	0.47	CSK	intronic
chr15:75086641	0.33	0.83	rs201978807	G	GGGTAA	0.05	0.33	0.13	0.42	CSK	intronic
chr15:75088962	0.91	0.99	rs11635664	G	T	0.04	0.39	0.18	0.59	CSK	intronic
chr15:75090349	0.95	0.99	rs12898997	C	T	0.03	0.39	0.17	0.6	CSK	intronic
chr15:75092384	0.6	0.94	rs2301249	T	C	0.58	0.56	0.57	0.69	CSK	intronic
chr15:75095483	0.61	0.94	rs7085	T	C	0.59	0.56	0.57	0.69	CSK	3'-UTR
chr15:75096443	0.6	0.94	rs1378938	T	C	0.5	0.56	0.58	0.69	903bp 3' of CSK	
chr15:75097780	0.91	0.98	rs35206230	C	T	0.03	0.39	0.17	0.59	2.2kb 3' of CSK	
chr15:75101530	0.92	0.98	rs34862454	C	T	0.03	0.39	0.17	0.6	3.5kb 5' of LMAN1L	

chr15:75102714	0.9	0.98	rs12591513	G	A	0.03	0.39	0.17	0.59	2.3kb 5' of LMAN1L	
chr15:75102851	0.92	0.98	rs12594062	T	C	0.03	0.39	0.17	0.6	2.2kb 5' of LMAN1L	
chr15:75102923	0.92	0.98	rs11630478	G	T	0.03	0.39	0.17	0.6	2.1kb 5' of LMAN1L	
chr15:75106138	0.58	0.92	rs4886619	C	T	0.57	0.56	0.58	0.69	LMAN1L	intronic
chr15:75107311	0.58	0.92	rs12441505	A	C	0.57	0.56	0.58	0.69	LMAN1L	intronic
chr15:75107880	0.57	0.9	rs7176022	A	C	0.56	0.56	0.57	0.69	LMAN1L	intronic
chr15:75114322	0.75	0.92	rs11636952	T	C	0.1	0.44	0.39	0.63	LMAN1L	intronic
chr15:75114374	0.57	0.91	rs11072509	C	G	0.57	0.56	0.57	0.69	LMAN1L	intronic
chr15:75115416	0.57	0.91	rs7180484	T	G	0.56	0.56	0.58	0.69	LMAN1L	intronic
chr15:75115895	0.57	0.91	rs7162232	G	A	0.57	0.56	0.58	0.69	LMAN1L	intronic
chr15:75116167	0.66	0.96	rs12917376	C	T	0.04	0.31	0.17	0.52	LMAN1L	intronic
chr15:75116184	0.58	0.91	rs11634474	C	G	0.56	0.56	0.58	0.69	LMAN1L	intronic
chr15:75117912	0.24	-0.91	rs79217743	G	T	0.02	0.13	0	0.16	LMAN1L	missense
chr15:75120211	0.56	0.9	rs3935077	C	G	0.57	0.55	0.58	0.69	RP11-414J4.2	intronic
chr15:75125645	0.54	0.87	rs6495122	A	C	0.2	0.48	0.18	0.52	1.5kb 3' of CPLX3	
chr15:75128501	0.45	0.82	rs9210	T	C	0.59	0.73	0.71	0.7	ULK3	3'-UTR
chr15:75129594	0.5	0.88	rs2290573	G	A	0.03	0.44	0.17	0.5	ULK3	intronic
chr15:75130015	0.29	0.56	rs36112106	AG	A	0.34	0.5	0.6	0.63	ULK3	intronic
chr15:75130093	0.36	0.64	rs12898397	T	C	0.1	0.54	0.35	0.58	ULK3	missense
chr15:75130573	0.36	0.64	rs2290572	A	G	0.37	0.56	0.35	0.58	ULK3	intronic
chr15:75131661	0.44	0.81	rs4886615	A	G	0.6	0.73	0.71	0.7	ULK3	synonymous
chr15:75131959	0.36	0.64	rs936227	A	G	0.38	0.56	0.34	0.58	ULK3	synonymous
chr15:75132164	0.45	0.82	rs936228	T	C	0.58	0.73	0.7	0.7	ULK3	intronic
chr15:75132319	0.45	0.82	rs936229	A	G	0.58	0.73	0.7	0.7	ULK3	intronic
chr15:75132490	0.36	0.64	rs12908814	C	G	0.38	0.56	0.34	0.58	ULK3	intronic
chr15:75135447	0.43	0.8	rs2290574	T	C	0.58	0.73	0.7	0.69	ULK3	5'-UTR
chr15:75136256	0.3	0.6	rs201476892	CA	C	0.15	0.55	0.51	0.56	SCAMP2	
chr15:75136257	0.43	0.74	rs6939	A	C	0.45	0.71	0.65	0.66	SCAMP2	
chr15:75136261	0.38	0.62	rs199794390	C	CG	0.09	0.59	0.51	0.6	SCAMP2	
chr15:75136261	0.44	0.7	rs6938	C	G	0.08	0.6	0.61	0.63	SCAMP2	
chr15:75136694	0.48	0.85	rs12487	T	C	0.03	0.3	0.07	0.51	SCAMP2	
chr15:75138801	0.37	0.67	rs200709622	AAAG	A	0.24	0.61	0.61	0.65	SCAMP2	intronic
chr15:75138802	0.37	0.66	rs201449809	AAG	A	0.25	0.61	0.61	0.65	SCAMP2	intronic
chr15:75138803	0.36	0.66	rs202061118	AG	A	0.27	0.61	0.62	0.65	SCAMP2	intronic
chr15:75138808	0.36	0.86	rs200440700	AG	A	0.05	0.38	0.18	0.43	SCAMP2	intronic
chr15:75139426	0.49	0.87	rs4480762	A	G	0.03	0.46	0.16	0.5	SCAMP2	intronic
chr15:75139696	0.62	0.8	rs12902690	A	G	0.09	0.57	0.5	0.61	SCAMP2	intronic
chr15:75140854	0.62	0.81	rs3765066	G	A	0.09	0.58	0.5	0.62	SCAMP2	intronic
chr15:75142040	0.48	0.8	rs4886613	G	A	0.3	0.71	0.7	0.67	SCAMP2	intronic
chr15:75145098	0.47	0.81	rs936230	T	C	0.54	0.73	0.71	0.68	SCAMP2	intronic
chr15:75147332	0.39	0.69	rs1869959	A	C	0.52	0.71	0.7	0.65	SCAMP2	intronic
chr15:75148773	0.47	0.8	rs7180432	A	G	0.32	0.72	0.7	0.68	SCAMP2	intronic
chr15:75153140	0.34	0.68	rs12906946	A	G	0.56	0.77	0.89	0.68	SCAMP2	intronic
chr15:75154779	0.34	0.67	rs6495124	C	T	0.59	0.77	0.9	0.67	SCAMP2	intronic
chr15:75155202	0.28	0.7	rs138821444	C	7-mer	0.04	0.43	0.23	0.47	SCAMP2	intronic
chr15:75155662	0.38	0.76	rs12900662	T	C	0.04	0.49	0.23	0.5	SCAMP2	intronic
chr15:75155896	0.44	0.85	rs11630918	C	T	0.03	0.47	0.16	0.48	SCAMP2	intronic
chr15:75157582	0.39	0.77	rs4643278	C	T	0.03	0.49	0.23	0.5	SCAMP2	intronic
chr15:75159433	0.37	0.75	rs11072511	A	G	0.32	0.56	0.28	0.51	SCAMP2	intronic
chr15:75160998	0.38	0.76	rs12902515	G	C	0.31	0.56	0.28	0.5	SCAMP2	intronic
chr15:75164530	0.38	0.76	rs4886608	G	A	0.3	0.56	0.28	0.5	SCAMP2	intronic
chr15:75165751	0.38	0.76	rs11857695	G	T	0.31	0.56	0.28	0.5	44bp 5' of SCAMP2	
chr15:75166335	0.44	0.85	rs12911254	G	A	0.02	0.47	0.16	0.48	628bp 5' of SCAMP2	
chr15:75167036	0.26	0.54	rs71411084	C	T	0.58	0.72	0.84	0.64	1.3kb 5' of SCAMP2	
chr15:75170082	0.38	0.76	rs12903205	A	G	0.32	0.56	0.28	0.51	4.4kb 5' of SCAMP2	
chr15:75170391	0.34	0.67	rs34852467	CG	C	0.62	0.75	0.89	0.67	4.7kb 5' of SCAMP2	
chr15:75173925	0.38	0.76	rs4886632	G	T	0.03	0.49	0.23	0.51	8.2kb 5' of SCAMP2	
