# Supplementary material for: Gene Silencing and Haploinsufficiency of Csk Increase Blood Pressure
Source: PLoS One. 2016 Jan 11;11(1):e0146841. doi: 10.1371/journal.pone.0146841 (PMC4713444; doi:10.1371/journal.pone.0146841)
Supplement: S3 Table — (PDF) [file pone.0146841.s004.pdf]

S3 Table. The catalog of Published Genome-Wide Association Studies for variants near the lead SNP (rs1378942, ± 1Mb boundary) (National Human Genome Research Institute).

PUBMED_ID	First Author	Year	Journal	Study	Disease/Trait	Strongest SNP-Risk Allele	Chr	Pos	Mapped_gene	p-Value	OR or beta	95% CI (text)
21876539	Amin N	2011	Mol Psychiatry	GWAS of coffee drinking suggests association with CYP1A1/CYP1A2 and NRCAM.	Coffee consumption	rs2470893-T	15	74727108	CYP1A1	2E-11	0.07	[0.05-0.09] unit increase
21490707	Cornelis MC	2011	PLoS Genet	GWAS identifies regions on 7p21 (AHR) and 15q24 (CYP1A2) as determinants of habitual caffeine consumption.	Caffeine consumption	rs2470893-T	15	74727108	CYP1A1	5E-14	0.12	[0.08-0.16] mg/day increase
21357676	Sulem P	2011	Hum Mol Genet	Sequence variants at CYP1A1-CYP1A2 and AHR associate with coffee consumption.	Coffee consumption	rs2472297-T	15	74735539	CYP1A1 - CYP1A2	5E-14	0.31	[0.17-0.44] increase in cups per day
21378988	The Coronary Artery Disease Genetics Consortium	2011	Nat Genet	GWAS in Europeans and South Asians identifies five new loci for coronary artery disease.	Coronary heart disease	rs2472299-?	15	74741059	CYP1A1 - CYP1A2	3E-6	NR	NR
21490707	Cornelis MC	2011	PLoS Genet	GWAS identifies regions on 7p21 (AHR) and 15q24 (CYP1A2) as determinants of habitual caffeine consumption.	Caffeine consumption	rs2472304-A	15	74751897	CYP1A2	3E-7	0.08	[0.06-0.10] mg/day increase
21909115	Ehret GB	2011	Nature	Genetic variants in novel pathways influence blood pressure and cardiovascular disease risk.	Diastolic/Systolic blood pressure	rs1378942-C	15	74785026	CSK	3E-26	0.42	[NR] mmHg increase
21909110	Wain LV	2011	Nat Genet	GWAS identifies six new loci influencing pulse pressure and mean arterial pressure.	Blood pressure	rs1378942-C	15	74785026	CSK	2E-15	0.39	[0.29-0.48] mmHg increase
19430483	Newton-Cheh C	2009	Nat Genet	GWAS identifies eight loci associated with blood pressure.	Diastolic blood pressure	rs1378942-C	15	74785026	CSK	1E-23	0.43	[0.35-0.51] mm Hg increase
21876539	Amin N	2011	Mol Psychiatry	GWAS of coffee drinking suggests association with CYP1A1/CYP1A2 and NRCAM.	Coffee consumption	rs6495122-A	15	74833304	CPLX3 - ULK3	7E-9	0.05	[0.03-0.07] unit decrease
21490707	Cornelis MC	2011	PLoS Genet	GWAS identifies regions on 7p21 (AHR) and 15q24 (CYP1A2) as determinants of habitual caffeine consumption.	Caffeine consumption	rs6495122-A	15	74833304	CPLX3 - ULK3	6E-7	0.07	[0.05-0.09] mg/day decrease
19430479	Levy D	2009	Nat Genet	GWAS of blood pressure and hypertension.	Diastolic blood pressure	rs6495122-A	15	74833304	CPLX3 - ULK3	2E-10	0.4	[0.28-0.52] mm Hg increase
23648065	Low SK	2013	Cancer Sci	GWAS of chemotherapeutic agent-induced severe neutropenia/leucopenia for patients in Biobank Japan.	Adverse response to chemotherapy (neutropenia/leucopenia) (carboplatin)	rs936229-G	15	74839978	ULK3	4E-6	1.69	[1.302-2.18]

rs2470893, rs2472297, rs2472299, rs6495122, and rs936229 are not in LD with the lead SNP (rs1378942).

rs2472304 is in LD with the lead SNP.
