# Supplementary material for: Gene Silencing and Haploinsufficiency of Csk Increase Blood Pressure
Source: PLoS One. 2016 Jan 11;11(1):e0146841. doi: 10.1371/journal.pone.0146841 (PMC4713444; doi:10.1371/journal.pone.0146841)
Supplement: S4 Table — (PDF) [file pone.0146841.s005.pdf]

S4 Table. Reduction of *Csk*, *Ulk3*, and *Cyp1a2* mRNA levels in cells after treatment with siRNAs.

siRNA Name	Target sequence (5'-3')	Reduction rate (%)	Cell line
Scrambled control siRNA	ACGUGACACGUUCGGAGAA		
Csk #1	GAAUGUAUUGCCAAGUACA	11.7	B16F10
Csk #2	ACAAGAAGUACGAAUCUUA	29.3	
***Csk* #3**	**CUGGCCAUCCGGUACAGAA**	**40.1**	
Ulk3 #1	UAUCUACCUCAUCAUGGAG	No reduction	B16F10
Ulk3 #2	ACGAAACAUCUCUCACUUGGA	73.4	
***Ulk3* #3**	**GGUUAUUUCUAAAGUUAGA**	**74.5**	
Cypla2 #1	CUGAACAUCGUGAAUAACA	22.1	NIH3T3
Cypla2 #2	GAUUGUCAACAUUGUCAAU	44.9	
***Cypla2* #3**	**GGUUUAAGACCUUCAAUGA**	**44.9**	
