# Supplementary material for: Gene Silencing and Haploinsufficiency of Csk Increase Blood Pressure
Source: PLoS One. 2016 Jan 11;11(1):e0146841. doi: 10.1371/journal.pone.0146841 (PMC4713444; doi:10.1371/journal.pone.0146841)
Supplement: S5 Table — (PDF) [file pone.0146841.s006.pdf]

S5 Table. Primers used for quantitative real-time PCR

siRNA Name	Forward primer (5'→3’)	Reverse primer (5'→3’)	product size (bp)
Gapdh	GCATGGCCTTCCGTGTTC	ATGTCATCATACTTGGCAGGTTTC	85
Cyp1a2	AGGGACACCTCACTGAATGG	GTCGATGGCCGAGTTGTTAT	150
Csk	TATCATGCGAGCAAGCTGAG	GCCACTGCGGTAGAACTCAT	165
Ulk3	GCTGGACGGTTTCATCCTTA	GTTTTCCACTGACGCCTTGT	145
